# Impact of Different Red Blood Cell Storage Solutions and Conditions on Cell Function and Viability: A Systematic Review

**DOI:** 10.3390/biom14070813

**Published:** 2024-07-08

**Authors:** Linh Nguyen T. Tran, Cristina González-Fernández, Jenifer Gomez-Pastora

**Affiliations:** 1 Department of Chemical Engineering, Texas Tech University, Lubbock, TX 79409, USA; nguyen-thuy-linh.tran@ttu.edu (L.N.T.T.); gonzalezferc@unican.es (C.G.-F.); 2 Chemical and Biomolecular Engineering Department, Universidad de Cantabria, Avda. Los Castros, s/n, 39005 Santander, Spain

**Keywords:** red blood cells (RBCs), storage solution, storage lesion, blood banking, storage conditions

## Abstract

Red blood cell (RBC) storage solutions have evolved significantly over the past decades to optimize the preservation of cell viability and functionality during hypothermic storage. This comprehensive review provides an in-depth analysis of the effects of various storage solutions and conditions on critical RBC parameters during refrigerated preservation. A wide range of solutions, from basic formulations such as phosphate-buffered saline (PBS), to advanced additive solutions (ASs), like AS-7 and phosphate, adenine, glucose, guanosine, saline, and mannitol (PAGGSM), are systematically compared in terms of their ability to maintain key indicators of RBC integrity, including adenosine triphosphate (ATP) levels, morphology, and hemolysis. Optimal RBC storage requires a delicate balance of pH buffering, metabolic support, oxidative damage prevention, and osmotic regulation. While the latest alkaline solutions enable up to 8 weeks of storage, some degree of metabolic and morphological deterioration remains inevitable. The impacts of critical storage conditions, such as the holding temperature, oxygenation, anticoagulants, irradiation, and processing methods, on the accumulation of storage lesions are also thoroughly investigated. Personalized RBC storage solutions, tailored to individual donor characteristics, represent a promising avenue for minimizing storage lesions and enhancing transfusion outcomes. Further research integrating omics profiling with customized preservation media is necessary to maximize post-transfusion RBC survival and functions. The continued optimization of RBC storage practices will not only enhance transfusion efficacy but also enable blood banking to better meet evolving clinical needs.

## 1. Introduction

Of the ~5 billion cells per milliliter of blood, red blood cells (RBCs) make up >99% of all cellular components, playing a central role in oxygen delivery throughout the body [1]. Most of the oxygen transport is carried out by the RBC protein hemoglobin (Hb), which comprises four subunits, each having one polypeptide chain and one heme group; each single Hb has four binding sites for oxygen in the iron (Fe) atoms present in the heme groups [2,3]. For many medical conditions, including certain types of anemia, RBC transfusions are required to supply the body with healthy RBCs and maintain a sufficient level of Hb [4] since vital organs, such as the heart, brain, and kidneys, have a limited ability to increase oxygen uptake during anemia, necessitating RBC transfusions to increase oxygen supply to the tissues [5].

Approximately 36,000 units of RBCs are used daily in the U.S., and hospitals have a great challenge to maintain a reliable supply, given the 6-week expiration period from the blood donation date [6]. Many hospitals have already amended their transfusion protocols since the Coronavirus Disease 2019 (COVID-19) pandemic and due to blood donor shortage by diluting RBC units (as well as tightening transfusion criteria) [6]. To maintain an appropriate supply of RBC units, RBCs obtained from healthy donors must be appropriately stored at blood banks because no widely available artificial oxygen-carrying substitutes exist to replace human blood [7]. Blood banks face several challenges in collecting and preserving donated RBCs to meet transfusion needs. Donations are limited due to the low number of eligible donors and restrictions on donation frequency to avoid adverse effects like donor anemia [8]. Therefore, research is directed toward improving storage techniques that can prolong the RBC unit’s shelf life to optimize donated blood utilization. Currently, the allowed storage duration is 42 days from donation. Enhancing RBC preservation efficacy and durability could expand blood reserves and reduce donor reliance. Refining RBC storage strategies is critical due to the lack of synthetic substitutes and persistent blood shortage and may result in the more effective use of the finite available supply of RBC units. Given the RBCs’ critical function, the effects of blood storage solutions and conditions on RBC viability and activity are substantial.

Different storage solutions have been developed over the last decades, like phosphate-buffered saline (PBS), different additive solutions (ASs), like AS-1, AS-3, AS-5, AS-7, and others, like SAGM, and PAGGSM, named after their constituents saline, adenine, glucose, and mannitol, and phosphate, adenine, glucose, guanosine, saline, and mannitol, respectively [9]. These contain preservatives to prolong the RBC shelf life, but an ideal storage solution has not been developed. For instance, PBS helps protect the RBC membrane integrity, but declined cell activity has been observed during prolonged storage [10]. SAGM preserves Hb levels but can increase free Hb accumulation [11]. Precisely, storage lesions, like declining adenosine triphosphate (ATP) and membrane integrity, compromise RBC functions and viability over time [12]. Moreover, RBC functions and viability are also affected by a multitude of storage factors, like pH, temperature, and changes in the solution composition [10,11]. For instance, changes in the pH can significantly affect RBC viability and stability [13]. On the other hand, other factors, like temperature, can influence the Hb levels and the protein activity [14,15]. Despite recent advancements in the development of improved storage solutions, limitations still remain. This review aims to address current challenges and to provide an overview of the progress in the field by presenting recently developed storage methods to minimize RBC storage-related lesions and maximize the RBC unit shelf life.

This review comprehensively analyzes various solutions and conditions employed for RBC unit storage, assessing their efficacy in maintaining viability and functions during hypothermic (1–6 °C) preservation. Key outcomes examined based on stored RBCs include ATP levels, the redox balance, membrane integrity, morphology, and Hb levels and stability, among others. By presenting and summarizing current evidence on different solutions and storage conditions, this review sheds light on the best practices for RBC storage that uphold cell integrity and function, minimizing potential side effects on transfusion recipients. Specifically, we aim to provide an exhaustive examination of the effects of storage solutions by assimilating pertinent research and evaluating both conventional and novel techniques to find optimal evidence-based approaches that minimize RBC storage lesions and maximize shelf life. The insights gathered from this work can inform blood-banking protocols, guide research directions for improving donated blood product preservation, and optimize real-world banking practices in this critical area.

## 2. Methodology

### 2.1. Search Strategy

A comprehensive systematic literature search following the Preferred Reporting Items for Systematic Reviews and Meta-Analyses (PRISMA) guidelines was conducted to identify as many as possible studies on RBC storage solutions, additives, and conditions. PRISMA provides an evidence-based protocol for conducting systematic reviews, including guidance on the optimal search strategies, study selection, data extraction, and reporting methodology. Using PRISMA enhances the thoroughness, consistency, and transparency of the review process. Comprehensive searches were conducted in PubMed, Web of Science, and Scopus. These databases were chosen because they cover novel research in biomedical and life sciences, ensuring a thorough search foundation for identifying relevant studies on RBC storage approaches. Search strategies combined controlled vocabulary terms (MeSH, Emtree), as well as free text keywords, such as “blood storage”, “red blood cell”, “erythrocyte”, “additive solution”, “hemolysis”, “metabolism”, “ATP”, “oxidative stress”, “cytokine”, “storage lesion”, and names of specific storage solutions. Reference lists of eligible studies were hand-searched. This rigorous, systematic search of multiple databases using both controlled and free-text terminology related to RBC storage maximized the identification of potentially relevant studies for inclusion in the review; thus, the comprehensive PRISMA-guided search methodology enhanced the scientific quality of this work.

### 2.2. Study Selection

**Figure 1:**
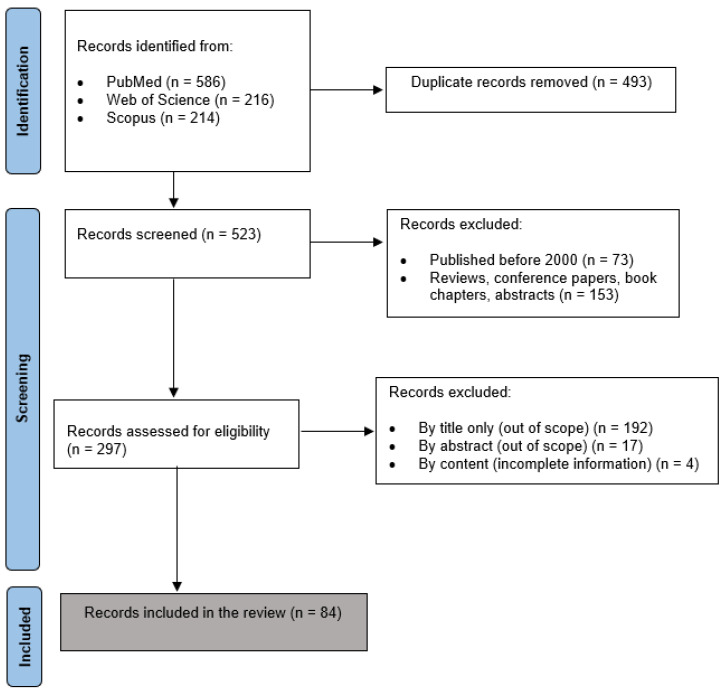
PRISMA flow diagram of the study selection process.

The selection of studies was executed meticulously, with a strong emphasis on the quality and scientific rigor of the published data [16]. In this context, we favored peer-reviewed original articles while excluding other publication formats, like short communications, technical reports, letters, notes, abstracts, and surveys. Unpublished works were also excluded to maintain the standard of trustworthiness. To incorporate only the most recent research in the field, only studies published between an inclusive timeline of 2000 and 2023 were finally incorporated in our analysis. The selection process involved deduplication, screening, and filtering stepwise. An initial search using the defined terms returned a substantial number of articles, the majority of which were unrelated to the focus of this work. The selection criteria focused on including only peer-reviewed journal publications, using the English language, and studies emphasizing how RBC storage solutions and conditions influence the properties and biological functions of the cells. A total of 84 studies were finally included in this work. This meticulous selection process was crucial in maintaining the quality and relevance of the studies included in our analysis. The study selection process is shown in Figure 1.

### 2.3. Data Extraction

A comprehensive, standardized data extraction form was created in Microsoft Excel 2021 (version 18.0) to systematically collect all relevant data from the included studies. Separate columns were designated in the spreadsheet to record study identification data, including the authors and publication year; sample size or number of blood units examined; storage solutions and additives (including the concentrations and composition of the solutions); duration of storage (usually ranging from 10 days to over 50 days); storage temperature (typically standard blood bank refrigeration of 1–6 °C); viability and functionality measures assessed via hemolysis, ATP, and 2,3 diphosphoglyceric acid (2,3-DPG) levels indicating metabolic status, cytokine levels, oxidative stress markers (protein carbonyls, oxysterols, isoprostanes, polyamines, and many more) [17,18], microvesicle formation, band 3 protein profiles (the most abundant protein in the RBC membrane responsible partly for membrane stability and functional regulation [18]), morphology, osmotic fragility, and others; time points at which the viability measures were assessed during storage; relevant statistical analyses performed and results; and author interpretations, conclusions, and discussions regarding the efficacy and performance of the different storage solutions and additives tested under the storage conditions examined. The standardized spreadsheet was populated to comprehensively extract and compile the relevant data from each of the 84 included studies in a consistent, unbiased manner. Careful data cleaning and quality control measures were implemented to ensure the accuracy and integrity of the final dataset. This methodology facilitated organizing the collected evidence on various RBC storage solutions, parameters, and storage durations for a comparative analysis to determine optimal approaches to maintain viability during prolonged, refrigerated RBC storage.

## 3. Storage Solutions and Their Measured Effect on RBC Storage

Over the years, various solutions have been developed with the goal of improving RBC storage and preservation. These formulations contain different relative concentrations of salts, sugars, buffers, and other constituents meticulously designed to address the challenges of extended RBC storage. The different components of the storage solutions can significantly impact the quality, safety, and efficacy of transfusions performed with the stored RBCs. Solutions are carefully designed to maintain RBC viability, prevent hemolysis, and ensure the effective oxygen delivery of stored RBCs when transfused. Currently, a wide variety of RBC storage solutions are available, each with unique compositions and attributes, as presented in Table 1. Having an array of options allows for customization to optimize RBC preservation and transfusion efficacy. Both inexpensive, straightforward solutions and more advanced additive media have been studied and utilized.

Extensive research has been conducted comparing these solutions to determine the optimal formulation for RBC storage. Table 2 presents a selection of key studies from 2000–2023 that analyzed and compared different RBC storage solutions and their resulting effects. By evaluating variables, such as the shelf life, biochemical changes, and post-transfusion recovery, these investigations provide insight into the relative merits of the available options. The continued comparative analysis serves to elucidate the ideal storage composition needed to balance prolonged preservation with the maintenance of cell quality and efficacy. Selecting the optimal solution remains imperative for ensuring positive outcomes for the transfusion recipients.

PBS is one of the earliest and most basic RBC storage solutions, first introduced in the 1950s. Comprised of a simple salt solution of sodium chloride, potassium chloride, and phosphate buffer, PBS provides some essential electrolytes to stored cells while maintaining osmotic balance [44]. The phosphate buffers help regulate pH by providing limited buffering capacity. However, the lack of glucose or other energy sources in PBS offers no nutrition to fuel RBC metabolism. While the balanced salts and osmotic environment help preserve the cells short-term, the limited composition of PBS cannot prevent metabolic derangements and acidosis during prolonged storage. Indeed, research has proven that PBS is restricted to a maximum RBC storage time of less than 1 week before substantial viability and functional loss occurs [27]. After this point, the inadequate buffering capacity and limited nutritional components of PBS fail to counteract accumulating biochemical and biomechanical storage lesions. Thus, while PBS continues to be widely used due to its low cost and ease of preparation, it has largely been replaced by more advanced ASs with additional compounds, as seen in Table 1, that enable extended storage while optimizing the preservation of RBCs. However, PBS may still provide a straightforward, inexpensive option for short-term storage needs of 1 week in duration.

While PBS provides a basic and inexpensive storage solution, its composition limits its use for extended preservation. This led to the development of the first AS in the 1960s, known as AS-1 (adenine-saline, commercially known as Adsol), which sought to build upon the simple PBS formulation by incorporating the key metabolite adenine [20]. Adenine helps maintain ATP levels to support basic metabolic functions during storage. AS-1 also contains glucose and mannitol, and the presence of mannitol in the AS-1 solution reduces hemolysis even in the presence of leukocyte proteases [42]. Even though the limited electrolytes and saline provide some osmotic balance, they cannot prevent metabolic derangements, oxidative damage, and cell swelling during prolonged hypothermic storage [45]. Research indicates that AS-1 leads to decreased ATP and 2,3-DPG levels, reduced oxygen offloading capacity, and increased oxidative lesions over time, as presented in some of the studies reported in Table 2 [46]. Specifically, the study of Meyer et al. demonstrated a 20% decrease in ATP levels of RBC units stored in AS-1 for 42 days [40]. With no additional nutritional or pH-modulating compounds, AS-1 allows for a maximum RBC storage time of up to 6 weeks before substantial viability and functional decline occurs [46]. After this, irreversible oxidative stress, vesiculation, and biomechanical changes accumulate without means to counteract them [46]. While offering a slight improvement over PBS, the minimalist composition of AS-1 prompted the development of more advanced solutions, such as AS-3.

The AS-3 solution (Nutricel) was developed in the 1980s as the next generation of ASs. As shown in Table 1, AS-3 builds upon the formulation of AS-1 by incorporating citrate and phosphate along with adenine and balanced salts, while omitting mannitol, which is present in AS-1. Additionally, AS-3 has been formulated to buffer intracellular pH by exploiting the chloride shift phenomenon through a low-chloride loading (70 mmol/L of NaCl in AS-3 vs. 154 mmol/L in AS-1) [20,38]. A high phosphate loading in AS-3 also provides a key substrate for salvage reactions for ATP biosynthesis [38]. However, storage in AS-3 is still associated with metabolic and structural changes over time that can impact RBC functions. For example, Rolfsson et al. found distinct metabolic profiles in AS-3-stored RBCs, characterized by decreased intracellular citrate and increased levels of metabolites [41]. The presence of glucose in this solution may also enhance oxidative damage through increased reactive oxygen species (ROS) generation during prolonged storage periods [30]. Additionally, the pH of AS-3 (around 5.8), while preventing severe acidosis, may accelerate the oxidative degradation of Hb and membrane proteins over extended storage compared to alkaline solutions [30]. Thus, AS-3 lacks some components to fully optimize long-term storage beyond 6 weeks without negative impacts on RBC function.

Although AS-3 can extend storage time compared to more rudimentary solutions, it is still limited in preventing RBC storage lesions beyond 6 weeks, as shown in the study of D’Amici et al. [30]. This spurred the development of the AS-5 solution (Optisol) in the 1970s as a further iteration on previous additive approaches [47]. AS-5 contains adenine, glucose, mannitol, and saline, as presented in Table 1. This composition is designed to maintain pH, control RBC swelling, and support cellular metabolism during its 42-day storage period [48]. The inclusion of mannitol, present in AS-5 and AS-1 but not in AS-3, serves as a free radical scavenger and provides membrane stabilization [30]. Additionally, the adenine in AS-5 helps mitigate the impact of adenine deaminase. While AS-5 improves cell preservation in comparison to earlier solutions, enabling RBC storage for up to 42 days, it still faces challenges in fully preventing storage lesions over this extended period. Like other ASs, AS-5 generates some level of oxidative damage during storage [48]. Despite its advancements, AS-5 lacks certain critical components for optimal prolonged storage without negatively impacting RBC functions. Therefore, and despite some incremental improvements over other storage solutions, AS-5 remains restricted to 6 weeks of storage before substantial lesions occur.

Capitalizing on AS-5’s progress, scientists developed the more advanced AS-7 solution (commercial name SOLX) in the 1990s seeking to further augment the RBC shelf life [49]. AS-7 is an advanced storage solution containing optimized adenine, mannitol, glucose, and electrolytes to prolong preservation to 6–7 weeks. The pH-balancing properties of mannitol prevent acidosis, while other components maintain the osmotic balance, metabolism, and ATP levels. This suggests that AS-7 can effectively protect against oxidative damage during storage. Nevertheless, AS-7 only slows but does not prevent the gradual depletion of metabolites, like ATP and 2,3-DPG, over time, indicating ongoing metabolic disturbances during storage. Structural changes, including membrane vesiculation, still occur with this solution, revealing that storage-related lesions continue to accumulate in AS-7 after 6–7 weeks of storage. Thus, while AS-7 constitutes an advance, it lacks the ability to prevent biochemical, biomechanical, and functional alterations.

AS-7 allows for RBC storage for up to 7 weeks through specialized nutrient additives. Seeking to build upon advancements, like AS-7, and further extend shelf life, next-generation solutions have been developed leveraging intracellular alkalinization, such as Erythro-Sol 5 (E-Sol 5) and phosphate-adenine-gluconate-guanosine-glucose-mannitol (PAG3M). Both induce sustained alkalinization by excluding chloride and incorporating cell-impermeant organic anions, like citrate (E-Sol 5) or gluconate (PAG3M), initiating the chloride shift mechanism that raises the intracellular pH [9]. Additionally, phosphate provides the substrate for ATP and 2,3 DPG generation. Compared to prior additives, RBCs stored in E-Sol 5 or PAG3M exhibit higher glycolysis rates, PPP activity, and ATP and 2,3 DPG preservation. Specifically, the study of Radwanski et al. [36] presented in Table 2 showed evidence of the chloride shift through elevated intracellular pH and concomitant decreased extracellular pH, increased RBC metabolism, and prolonged 2,3-DPG maintenance of RBCs when stored in E-Sol 5. Additionally, the gluconate-containing storage solution PAG3M has proven to be the only solution showing significant positive correlations between the levels of ATP and 2,3-DPG, highlighting a metabolic peculiarity of RBCs stored in the presence of the PAG3M formula [9]. Thus, through specialized formulations and induced intracellular alkalinization, these next-generation solutions aim to build on prior advancements to further prolong the RBC shelf life while maintaining metabolic functions and integrity.

While SAGM (saline-adenine-glucose-mannitol) has been extensively used to extend RBC storage for 6 weeks, recent research suggests that it may not be the most effective solution for maintaining key molecules, like ATP and 2,3-DPG [20,23]. Developed in the 1990s, SAGM aimed to optimize storage with its blend of salts, sugars, and other components, as outlined in Table 1. These ingredients aim to maintain osmotic pressure, provide metabolic fuel, support ATP production, and regulate pH. Additionally, chloride salts and antioxidants were included for protection from oxidative damage. Although SAGM can extend the RBC shelf life to at least 6 weeks, some works have reported that it may not maintain higher levels of ATP and 2,3-DPG compared to newer solutions, like PAGGGM or AS-7 [23,25,35]. Additionally, metabolic and oxidative changes continue to accumulate during storage with SAGM. One potential drawback of SAGM is its acidic pH, which can contribute to protein and membrane damage over long storage periods. Thus, while SAGM offers some improvements in RBC preservation, storage lesions still occur highlighting opportunities to further optimize the additives and buffering capacity for storage beyond 6 weeks.

A novel RBC storage solution called MAP (mannitol-adenine-phosphate) has been developed to better maintain cell functions and morphology during hypothermic storage. MAP provides energizing substrates, like glucose, adenine, and phosphate, to fuel RBC metabolism via glycolysis and the hexose monophosphate shunt [43]. By preserving ATP, MAP also maintains RBC deformability, which relies on the ATP-dependent calcium pumping and membrane PL asymmetry [43]. Compared to saline, MAP better retains the discoid morphology and deformability throughout storage and washing-associated RBC damage and destruction. While the precise mechanisms are still being elucidated, it is hypothesized that the impermeant sugar mannitol in MAP may also protect against oxidative damage during processing [43]. Overall, through tailored nutrient additives that boost metabolism and help maintain membrane integrity, MAP prevents functional and morphological lesions induced by standard cell washing protocols—improving RBC quality during storage.

As research continues to advance RBC storage solutions, a promising novel option is PAGGSM. This solution builds on prior formulations but incorporates additional key compounds to further optimize RBC preservation, such as guanosine. The phosphate buffers of this solution help regulate the pH to prevent acidosis, while mannitol provides osmotic balance and prevents excessive cell swelling. Glucose supplies the necessary energy substrate to fuel metabolic processes, and adenine supports ATP generation. Uniquely, PAGGSM also contains guanosine, which aids in the synthesis of protective guanosine triphosphate. This component has antioxidant properties that can help counteract oxidative damage during storage. Together, these ingredients create an optimized storage environment that counteracts damaging acidosis, oxidative stress, and harmful biomechanical changes. Published works showed that PAGGSM can extend the RBC shelf life to over 42 days, while maintaining higher ATP levels and limited hemolysis compared to other solutions [23,25,35]. Moreover, PAGGSM enables improved oxygen-delivery capacity after prolonged storage compared to other ASs [9].

As presented in Table 2, an important parameter to follow during storage to determine appropriate storage conditions is adequate gas diffusion, which is an essential function of RBCs. However, balancing permeability with osmosis remains challenging [50]. Maintenance of the optimal pH and electrolyte gradients also impacts gas transport and exchange. Although solutions like PAGGSM better maintain oxygenation in comparison to other options, like AS-1, deficiencies still alter metabolism [50].

Sufficient nutrient availability is also critical to support RBC viability during prolonged storage. Providing key nutrients, like glucose, adenine, and guanosine, fuels RBC metabolism to maintain functions during storage. However, the buildup of toxic metabolic intermediates must also be prevented over time. Available data presented in Table 2 indicate that PAGGSM and AS-7 optimally sustain nutrient availability to maintain the metabolism, energy status, oxygen delivery, and biosynthesis in the RBC units [23]. Additionally, the research by De Bruin et al. [25] revealed that PAGGSM had lower glucose utilization than SAGM and AS-3 over 6 weeks. These data suggest that PAGGSM and AS-7 better retain nutrients, while AS-1, AS-3, SAGM, and PBS demonstrate depletion during storage time.

Moreover, PAGGSM and AS-7 can also optimize oxidative stress control and membrane preservation during storage. Managing oxidative damage and preserving membrane structural stability are both essential during prolonged hypothermic RBC storage. Oxidative stress directly damages membrane components, like lipids and proteins. Even minor oxidation over time can lead to protein denaturation, lipid peroxidation, and cytoskeletal disruption [9]. Recent investigations indicate that PAGGSM and AS-7 best limit oxidative stress while maintaining membrane integrity in stored RBCs [23,37,49].

Complementing membrane integrity, Hb stability is also a key factor during prolonged hypothermic storage due to the function of this protein in O_2_ transport throughout the body. Limiting Hb oxidation and denaturation during storage preserves the oxygen delivery capacity after transfusion. However, prolonged hypothermic conditions can enhance instability over time. Assessing methemoglobin (metHb) levels, heme loss, and denatured Hb could identify solutions that better stabilize Hb [9]. Accumulated evidence suggests that AS-3, AS-5, AS-7, and PAGGSM most effectively maintain Hb stability during prolonged storage, as presented in Table 2 [19,24,29,37]. These data indicate that solutions like AS-3, AS-5, AS-7, and PAGGSM can be used to optimize Hb preservation in RBC units.

Finally, knowledge about how long RBC units may be safely stored is a crucial consideration for the reliability of the RBC unit supply. Several groups have explored storage time using several storage solutions, especially AS-3. According to published research, RBCs can be retained in AS-3 for up to 42 days if the solution is kept at 4 °C [48]. However, other solutions, like PAGGSM and AS-7, have shown promising results in maintaining nutrient availability, controlling oxidative stress, and preserving membrane integrity during longer storage periods of up to 56 days [23]. These findings highlight that while current solutions offer extended storage times, there is still room for improvement. Additionally, storage can be enhanced and extended by modifying the solution parameters, which is the focus of the next section.

## 4. Other Preservation Strategies for Enhancing RBC Storage

Beyond storage solutions, other RBC preservation parameters, like the pH, temperature, oxygenation, anticoagulants, and presence of other molecules and contaminants, can impact RBC viability during refrigerated storage. The pH affects protein structure and enzyme functions, and the storage temperature regulates metabolic processes [51,52]. The oxygen content requires careful control to balance supply and oxidative damage. Anticoagulants differ in maintaining RBC membrane integrity. The level of contaminants may accumulate reactive byproducts, impairing cells. The optimization of these secondary factors represents a complementary approach to enhance RBC quality and minimize storage lesions [53]. In this section, we introduce the effects of these conditions, as well as some of the most relevant recent studies that have been published in the literature (see Table 3).

Table 3 summarizes critical studies investigating the roles of modifying the pH, temperature, oxygenation, and anticoagulants (among others) in RBC preservation outcomes. This provides an evidence-based guide to multifaceted blood storage strategies maximizing viability and function. Indeed, the maintenance of an alkaline pH throughout prolonged storage limits metabolic abnormalities and improves morphology. However, optimal recovery and functionality post-transfusion require balancing multiple factors beyond just pH optimization. The current, conventional RBC storage solutions are adjusted to a pH of 5.5–5.8, which is well below the physiological pH of 7.4 [83]. Within this pH range, RBCs utilize their natural buffering capacity to counteract the buildup of lactic acid and other metabolites. However, below a pH of 6.5, viability and function decline more rapidly. Recent studies have explored the impact of maintaining a higher pH throughout storage to extend the shelf life of RBC units [20]. They found that units stored in alkaline PAGGGM had higher ATP and 2,3-DPG levels, a normal morphology, and lower hemolysis compared to conventional storage [25,84]. However, the PTR of these alkaline-stored RBCs was no different than the conventional units. This approach could extend the tolerable storage duration while utilizing existing standard-of-care solutions. Collectively, the maintenance of a more alkaline pH during prolonged RBC storage has physiological benefits in terms of metabolism, morphology, and hemolysis markers [9].

While preserving an alkaline pH during extended storage mitigates metabolic derangements and morphological anomalies, attaining favorable clinical outcomes after transfusion necessitates optimizing multiple elements, not just pH. Another pivotal factor is storage temperature. The standard practice of 1–6 °C refrigeration enables practical blood banking, even though storing at room temperature could offer logistical benefits [50]. However, decreasing the temperature below 0 °C might not offer additional advantages. A recent study by William et al. [50] explored the utility of extracellular additives to mitigate damage during experimental hypothermic storage at −4 °C. Storing RBCs at this reduced temperature alone exacerbated the typical storage-induced decline in deformability and increase in hemolysis compared to 4 °C, despite beneficially retaining more ATP. Additionally, Eckstein et al. [71] investigated the impact of a 24 h room temperature hold (RTH) prior to rejuvenation and subsequent refrigerated storage on the RBC integrity. Compared to rapidly cooled (RC) units, RBCs subjected to RTH initially showed reduced potassium and 2,3-DPG levels but conventional assessment markers like hemolysis and morphology were unaffected. Therefore, while refrigeration remains essential for current blood banking viability, both hypothermic enhancement and transient ambient-damage ramifications demand additional meticulous exploration as storage science continues advancing.

The choice of anticoagulants impacts the preservation quality of stored RBCs intended for transfusion. Burger et al. [79] compared RBC units collected and stored in different anticoagulant/AS combinations over 5 weeks. Units collected in standard CPD anticoagulant and stored in conventional SAGM showed substantially worsened ATP maintenance by 35 days in comparison to units collected in TNC and stored in experimental PAGGGM solutions. The acidic CPD conditions (pH 5.6) likely contributed to a different metabolism and storage lesion accumulation versus the more physiologic TNC pH of 7.4. Moreover, the TNC and PAGGGM formulations specifically aimed to optimize the pH buffering capacity. Burger et al. [79] also prepared PAGGGM solutions at both physiologic (7.4) and alkaline (8.2) pH from TNC-collected blood, finding no significant differences. Thus, starting with a physiologic anticoagulant pH enabled reducing the storage solution pH without sacrificing beneficial metabolic preservation. On another hand, Meledeo et al. [77] recently assessed the coagulation function in whole blood units collected with CPD, CP2D, and CPDA-1 anticoagulants over 5 weeks of refrigerated storage. Surprisingly, most parameters showed minimal deterioration beyond 21 days across all groups. Specifically, the RBC count, HCT, and Hb level did not significantly decline over time in any anticoagulant. Additionally, the RBC count was significantly different between CPD and CP2D on day 3 but not at any other time point. In all three anticoagulants, the pH declined significantly over the storage duration, likely due to an observed 10-fold increase in lactate (and complementary reduction in bicarbonate). CP2D units displayed greater derangements for the lactate, bicarbonate, and base deficit by 2 weeks. No exclusive detriments appeared in the CPD/CP2D units by 3 weeks versus CPDA-1 units at 5 weeks. They also found that a fresher blood sample provided greater hemostatic functionality, particularly over the first 2 weeks, but these studies show no reason for CPD/CP2D to be restricted to a shorter shelf-life than CPDA-1. Therefore, anticoagulant selections significantly, and often undeservedly, dictate acceptable storage durations. Clarifying the evidence-based limits for deterioration could expand the usage of extended-preserved products, potentially improving the availability and quality of blood products for transfusion.

The oxygenation state of the storage environment can significantly influence metabolic processes and oxidative damage accrual affecting RBC quality over time. While original preservative solutions utilize atmospheric oxygen tensions, anaerobic storage below 20 mmHg is now becoming more common to mitigate oxidative threats. However, optimal targets balancing benefits and feasibility remain debated. Meng et al. [85] recently explored replacing air with helium to create hypoxic erythrocyte storage. This effectively reduced dissolved oxygen by 75% and pO_2_ five-fold versus air-equilibrated controls throughout storage. Despite consuming more glucose and ATP, hypoxic cells better maintained 2,3-DPG levels. Hemolysis remained under 0.8% at 9 weeks, meeting quality thresholds, while controls exceeded specifications. Though deformability and osmotic fragility changes were comparable, hypoxic cells had reduced PS exposure indicating less membrane oxidative damage. Critically, over half of the hypoxic RBCs retained reversible discocyte morphology by week 9, whereas controls showed predominantly irreversible spherocytosis. D’Alessandro et al. [86] meanwhile found that anaerobic storage uniquely enabled the intact maintenance of metabolic and redox homeostasis. Anaerobic cells displayed sustained glycolytic fluxes and 2,3-DPG, decreased lipid oxidation, preserved glutathione and protein thiol, and retained the ATP and antioxidant capacity. Oxygenated storage severely depleted glutathione and ATP by 6 weeks as oxidant lesions accumulated. In summary, ambient aerobic storage permits escalating oxidative damage over time. Anaerobic and hypoxic conditions prevent ROS accumulation, conferring functional preservation advantages. However, anaerobic storage poses logistical tradeoffs, while hypoxic storage largely averted storage-associated derangements. Nevertheless, limiting oxidant exposure through reduced oxygenation emerges as a pivotal strategy for attenuating storage-related injury.

While oxygen minimization helps mitigate oxidative damage, the progressive accumulation of metabolic dysfunction also intrinsically correlates with the length of hypothermic RBC storage itself. Current 42-day limits attempt to balance availability, economics, and safety [10]. However, several countries mandate shorter 35-day thresholds given accumulating indications [87]. Product integrity measurably deteriorates over prolonged hypothermic maintenance. Even though defining optimal storage durations remains an open question, the specific milieu within which prolonged hypothermic preservation unfolds profoundly influences the pace of accrued metabolic anomalies and functional decays. The compositional properties of supplementary storage media emerge as pivotal tools for incrementally mitigating the multifaced storage lesion progression [57].

In addition to the listed effects, several other factors can significantly impact RBC quality during hypothermic preservation. One such consideration is irradiation. Gamma or x-ray irradiation helps prevent transfusion-associated graft versus host disease by inactivating residual lymphocytes in cellular blood products before transfusion [88]. However, multiple studies have indicated that irradiation exacerbates free radicals and ROS can damage RBC membranes and worsen metabolic dysfunction. Sparrow et al. [20] found that irradiating RBCs dramatically increased hemolysis, potassium leakage, PS exposure, and vesiculation after 2 weeks of storage compared to unirradiated controls. Meanwhile, irradiation after just 3 days of storage minimally impacted in vitro measures, though in vitro 24 h recoveries declined 10% regardless of timing. Thus, pre-storage leukoreduction helps avoid irradiation-related quality decays in stored RBCs [89]. When irradiation is necessary, treating the units as early as possible, preferably before storage, mitigates deleterious effects on overlaying storage lesion accumulation [89].

Washing and rejuvenating old blood units have also been explored as a means to partially reverse storage-related changes before transfusion. Rejuvenation refers to incubation with metabolic substrates and cofactors to regenerate ATP and 2,3-DPG, reverse membrane changes, and restore NO scavenging capacity and deformability [30,81]. Tchir et al. [61] found that a cold rejuvenation of RBCs during hypothermic storage increased intracellular ATP, but this change did not ameliorate, or exacerbate, the metabolic or biochemical symptoms of the storage lesion. Meanwhile, washing younger units can reduce pro-inflammatory bioactive metabolites, like lipids, iron, and cytokines, that accumulate by later storage phases [67]. Still, the clinical benefits of these approaches remain uncertain.

Maintaining the sterility of stored blood is also essential. Pathogen reduction methods, like the INTERCEPT Blood System, help prevent bacterial or viral transmission risks. A study by Dimberg et al. [82] examined the impacts of the Mirasol pathogen reduction technique on stored RBCs. While ATP levels and other in vitro parameter levels remained unaffected compared to non-treated controls, Mirasol treatment did significantly increase potassium levels, which worsened further over the storage time. Interestingly, metHb in Mirasol-treated samples returned to normal levels within 24 h of treatment, even though it was high on day 0. The study suggested that RBCs derived from Mirasol-treated samples are suitable for transfusion throughout 21 days of storage. Thus, while ensuring sterility, Mirasol intensified the biochemical and morphological expressions of the storage lesion.

Finally, and although not fully considered in the literature and in this work, it should be noted that intrinsic genetic and environmental donor variables also influence baseline RBC properties in ways that may affect storage lesion progression. Parameters like the donor age, gender, diet, and lifestyle can affect the RBC status at collection and during subsequent refrigeration. For example, a study by Mykhailova et al. [63] reported that RBCs from females contribute less to the storage lesion and age slower than males’ RBCs. Nevertheless, a different study reported that females tend to donate blood units with lower Hb levels, an effect also seen in older donors and individuals with lower body weight [90].

Overall, while incremental advancements continue, storage solutions remain inadequate to fully arrest storage progressive derangements. Further efforts to integrate multi-omics profiling illuminating patient-attributable fragilities with customized, stabilized media are imperative to shift from generalized population standards toward personalized optimization maximizing post-transfusion circulatory fitness and survival.

## 5. Conclusions and Further Research

RBC storage solutions have advanced considerably over the last decades, evolving from early basic formulations, like PBS and AS-1, to more optimized options, like SAGM and PAGGSM, that better maintain cell integrity and functions during prolonged hypothermic preservation [10,44]. Key storage parameters include the pH-buffering capacity to prevent acidosis, the osmotic balance, metabolic substrates to support RBC metabolism and ATP generation, antioxidants like AA and uric acid to counter oxidative threats, and electrolyte blends sustaining transport and volume regulation [44,45,52,91,92,93]. First-generation solutions lacked comprehensive components, like PBS, restricting storage to 1 week before substantial lesions occur. Second-generation options added key ingredients enabling 4–6-week stability, while third-generation solutions further optimized constituents to prolong storage to 8 weeks without significant adverse impacts [38,46]. These latest generation solutions leverage intracellular alkalinization or tailored supplementation to enable reliable 8-week storage with preserved structural morphology, gas diffusion, and transfusion capacity [9,19,23,36,38,44]. Recent comparative studies of these various storage solutions provide valuable insights into their relative efficacies in preserving RBC quality during storage [23,94].

However, despite incremental advancements, current solutions can only partially prevent time-dependent biochemical, biomechanical, and functional decay [44]. Metabolic anomalies, oxidative damage, and morphological changes still accumulate by later storage phases across present formulations [45]. Therefore, continued efforts to clarify multi-scale mechanisms underlying progressive storage fragility combined with solutions tailored to match discrete deficiency profiles offer paths toward personalized optimization, maximizing post-transfusion circulatory fitness and survival [44]. Hence, while incremental advancements have extended achievable storage limits, truly optimized solutions require personalized profiling coupled with targeted stabilization countermeasures.

While this review has primarily focused on refrigerated storage methods for RBCs in a liquid form, it is important to acknowledge the significance of frozen RBC storage. Recent global events, including the COVID-19 pandemic, have highlighted the potential benefits of more long-term preservation techniques using alternative, frozen storage methods [95,96]. Future research efforts should aim to comprehensively compare liquid refrigeration and frozen storage methods, evaluating their respective advantages, limitations, and impacts on RBC quality and functionality over extended periods. Such comparative studies could provide crucial insights for optimizing blood banking practices, potentially extending the RBC shelf life while maintaining cellular integrity and function. This approach aligns with the broader goal of developing more personalized and efficient storage strategies.

Further research should focus on elucidating donor-specific differences in baseline RBC properties and storage lesion progression rates utilizing multi-omics profiling correlated to clinical outcomes. These data can inform the development of tailored storage solutions catering to inherent weaknesses based on identifiable molecular patterns linked to factors like donor age, sex, genetics, lifestyle, and disease status [97,98]. Research should also continue to investigate the complex interplay between storage duration, temperature, oxygenation, anticoagulants, irradiation, handling methods, and intrinsic storage media properties in driving storage-related decay [45]. Incrementally mitigating identified deleterious changes through multi-factor optimization could better maintain homeostasis. Finally, another critical need is developing rapid and cost-effective analysis and tests gauging RBC structural integrity, metabolic fitness, oxidative resilience, and gas diffusion capacity to enable standardization of acceptable quality benchmarks for stored units before release. Ultimately, these multifaceted research efforts will revolutionize blood banking practices, ensuring the highest quality and longest shelf life of stored RBCs.

## Figures and Tables

**Table 1:** Composition of most common RBC storage solutions [19,20,21,22,23]. The table presents the composition of phosphate-buffered saline (PBS), various additive solutions (ASs), like AS-1, AS-3, AS-5, AS-7, Erythro-Sol 5 (E-Sol 5), saline, adenine, glucose, and mannitol (SAGM), phosphate, adenine, glucose, guanosine, saline, and mannitol (PAGGSM), phosphate, adenine, gluconate, guanosine, glucose, mannitol (PAG3M), and mannitol, adenine, phosphate (MAP).

Constituents (mmol/L)	PBS	AS-1	AS-3	AS-5	AS-7	E-Sol 5	SAGM	PAGGSM	PAG3M	MAP
**NaCl**	137	154	70	150	—	—	150	72	—	85
**NaHCO_3_**	—	—	—	—	26	—	—	—	—	—
**Na_2_HPO_4_**	10	—	—	—	12	20	—	16	8	—
**NaH_2_PO_4_**	—	—	23	—	—	—	—	8	8	6
**Gluconate**	—	—	—	—	—	—	—	—	40	—
**Citrate**	—	—	2	—	—	25	—	—	—	1
**Na-Citrate**	—	—	23	—	—	—	—	—	—	5
**Adenine**	—	2	2	2.2	2	2	1.25	1.4	1.4	1.5
**Guanosine**	—	—	—	—	—	—	—	1.4	1.4	—
**Glucose**	—	111	55	45	80	111	45	47	47	40
**Mannitol**	—	41	—	45.5	55	41	30	55	55	80
**KCl**	2.7	—	—	—	—	—	—	—	—	—
**KH_2_PO_4_**	1.8	—	—	—	—	—	—	—	—	—
**pH**	7.4	5.5	5.8	5.5	8.5	8.4	5.7	8.2	8.2	5.7
**Storage Duration**	<1 week	42 days	42 days	42 days	56 days	56 days	42 days	42 days	56 days	42 days

**Table 2:** Effects of different storage solutions on RBC preservation. These studies analyzed and compared different storage solutions (first column) and their effect on RBC storage (fourth column).

Storage Solution Tested	Anticoagulant	Parameters Measured	Main Results	Storage Time	Reference
PAGGSM, SAGM	—	Mean corpuscular volume (MCV), hematocrit (HCT), morphology, hemolysis, osmotic fragility, echinocytes, RBC aggregability, deformability, and viscosity.	While hypertonic SAGM better prevented initial RBC swelling, isotonic PAGGSM showed advantages in maintaining RBC integrity throughout storage. Both solutions had similar effects on RBC shape, deformability, aggregability, and blood viscosity.	42 days	[24]
SAGM, PAGGGM	—	Posttransfusion recovery (PTR), metabolic restoration after transfusion (glucose, redox metabolism), and RBC phenotype.	There was no difference between the PTR of SAGM- and PAGGGM-stored RBCs. Glucose and redox metabolism were better preserved when stored in PAGGGM.	35 days	[25]
SAGM	Citrate phosphate dextrose adenine (CPDA)	Lactate dehydrogenase (LDH), lactate levels, potassium, HCT, sodium, glucose, and MCV.	CPDA RBCs developed storage lesions faster than SAGM-stored cells. Initially, samples in CPDA had higher potassium, lactate, HCT, and LDH levels, and lower sodium, MCV, and glucose levels compared to SAGM. After irradiation, the trends persisted.	28 days	[26]
SAGM, PAGGSM, SOLX, E-Sol 5, PAG3M	—	ATP levels, hemolysis, osmotic fragility, phosphatidylserine (PS) exposure, morphology, 2,3-DPG, MCV, pH, echinocytes, guanine, inosine, and adenosine levels.	Alkaline additives, especially PAG3M, better preserved 2,3-DPG and ATP levels in stored RBCs. SAGM had the highest MCV and osmotic fragility. PAG3M and PAGGSM maintained a lower pH than SOLX and E-Sol 5. E-Sol 5 and PAG3M had the least hemolysis and slightly higher PS exposure. All additives showed comparable echinocyte percentages. PAG3M best preserved 2,3-DPG levels, followed by SOLX and E-Sol. Guanosine supplementation in PAGGSM and PAG3M increased guanine and adenosine levels and decreased inosine.	56 days	[9]
SAGM, PBS	—	Composition, size, and concentration of extracellular vesicles (EVs).	RBCs stored in PBS suffered from vesiculation and RBC EVs had high Hb concentration content.	42 days	[27]
SAGM, AS-1	—	Morphology, RBC size, pH, hemolysis, glycophorin A (GPA) and PS EV quantitation, and endothelial cell (EC) interaction.	AS-1-stored RBCs had more irregular cell surfaces, lower hemolysis, higher MCV, and fewer GPA, PS EVs, and decreased adherence to ECs than SAGM-stored RBCs. The pH declined in both solutions but stayed above 6.5.	42 days	[28]
AS-3, AS-1	—	RBC product mass, HCT, Hb concentration, RBC count, pH, free Hb level, 2,3-DPG, ATP, glucose, sodium, potassium, lactate, hemolysis, osmolality, and morphology.	The in vitro and in vivo characteristics of the RBCs were satisfactory after deglycerolization and extended storage. The RBC recovery after deglycerolization was >93% for both AS-1 and AS-3 units. All RBC units had less than 1% hemolysis.	15 days	[29]
SAGM, AS-3	—	Membrane protein profile.	AS-3-stored RBCs had a better membrane protein profile than SAGM-stored RBCs. Membrane protein fragmentation is common during RBC storage, as evidenced by the increase in fragmentation spots at day 21 and significant decrease in fragmentation spots at day 42. Both AS-3 and SAGM failed to efficiently prevent protein fragmentation during RBC storage.	42 days	[30]
SAGM	—	Rheology, aggregability, osmotic fragility, deformability, ATP levels, MCV, pH, and mean corpuscular hemoglobin concentration (MCHC).	RBC aggregability decreased in the first week but recovered in the following weeks. Osmotic fragility and deformability were not significantly affected. ATP levels decreased by 54% during a 5-week storage period. MCV, pH, and MCHC remained relatively stable with minimal changes.	49 days	[31]
SAGM, PAGGSM, PAG3M, E-Sol 5, AS-7	Citrate-phosphate-dextrose (CPD)	Metabolites (ATP, 2,3-bisphosphoglycerate (2,3-BPG), etc.), hemolysis, and morphology.	RBCs in PAG3M, E-Sol 5, and AS-7 had higher lactate production, ATP, and total adenylate levels. 2,3 BPG levels decreased in SAGM and PAGGSM units but were maintained or increased in E-Sol 5, AS-7, and PAG3M. Hemolysis was similar for all ASs. The newer ASs (PAGGSM, PAG3M, E-Sol 5, and AS-7) better preserved the morphological properties of RBCs compared to SAGM.	56 days	[23]
AS-1, pyruvate–inosine–phosphate–adenine (PIPA)	—	p50 (partial oxygen pressure at 50% oxygenation), deformability, hemolysis, and fragility.	Cold and standard rejuvenation with PIPA restored the oxygen-carrying capacity (p50) of RBCs without increasing hemolysis. Rejuvenation increased RBC deformability.	15 days	[32]
AS-7, AS-1	—	pH, glucose, lactate, bicarbonate, ATP, potassium, hemolysis, morphology, EVs, and PTR.	AS-7 reduced hemolysis, microvesicle release, and improved 24 h PTR compared to AS-1. AS-7 units had higher ATP levels.	56 days	[19]
SAGM	CPD	ATP level, pH, cation homeostasis, oxidative stress, morphology, EVs, and phospholipid (PL) composition.	RBCs maintained their PL content, despite abundant vesicle formation. RBCs and EVs had similar PL profiles, with no accumulation of raft lipids (e.g., cholesterol and sphingolipids) in RBC EVs. EV PLs had shorter acyl chains.	36 days	[33]
SAGM	CPD	Extracellular potassium, internal and external pH, calcium levels, ATP, oxidative stress, and hemolysis.	Nutrient levels in the storage solution decreased, while potassium and calcium levels within the cells increased. ATP levels declined, glutathione homeostasis got disrupted, oxidative stress and lipid damage increased, resulting in metabolite buildup.	42 days	[34]
SAGM, PAGGGM	CPD-50	ATP, 2,3-DPG, glucose consumption, lactate production, intracellular and extracellular pH, potassium concentration, and hemolysis.	RBCs stored in PAGGGM had higher 2,3-DPG and ATP levels compared to SAGM, despite having a similar intracellular pH throughout storage. During early storage, PAGGGM had higher glucose consumption, lactate production, fructose-1,6-diphosphate, and dihydroxyacetone phosphate levels due to increased phosphofructokinase (PFK) activity. PFK activity decreased in PAGGGM after 21 days, but sufficient metabolic reserve prevented the depletion of 2,3-DPG and ATP. The higher PFK activity in the first weeks of storage in PAGGGM compared to SAGM was likely responsible for preventing the depletion of 2,3-DPG and ATP.	35 days	[35]
E-Sol 5, AS-1	—	RBC counts, pH, glucose, potassium, phosphate, free Hb, ATP, 2,3-DPG, lactate levels, RBC morphology, and microparticles.	RBCs stored in E-Sol 5 had lower hemolysis, fewer microparticles, and better morphology compared to AS-1. E-Sol 5 demonstrated a chloride shift, with increased intracellular pH, decreased extracellular pH, increased cell metabolism, and 2,3-DPG preservation. E-Sol 5 slowed the progression of storage lesions.	42 days	[36]
SAGM, Erythrosol-4, PAGGSM	CPD	Morphology, microparticles, osmotic fragility, the adhesion of RBCs to the endothelium, and Hb levels.	RBCs stored in Erythrosol-4 and PAGGSM had decreased cell size, reduced osmotic fragility, and decreased accumulation of GPA in microparticles and annexin V-binding microparticles compared to RBC stored in SAGM. An increase in adherence to the endothelium was seen in RBCs stored in Erythrosol-4.	49 days	[37]
AS-3, AS-7	Citrate phosphate double dextrose (CP2D) or CPD	RBC indices and metabolomic analysis.	AS-3 and AS-7 had similar metabolic trends over storage. AS-7 stored RBCs showed higher metabolic activity in the first week, with increased pentose phosphate pathway (PPP) activity, higher glutathione levels, and elevated glycolysis.	42 days	[38]
SAGM, AS-1	CPD	PS exposure, microparticles, glutathione levels, and oxidative stress.	SAGM stored RBCs showed higher levels of PS exposure and released more EVs and more PS-positive EVs when subjected to transfusion stress compared to AS-1 units.	35 days	[39]
AS-1, AS-3, AS-5	CPD and CP2D	2,3-DPG and ATP levels.	No significant difference in the response decline among the three ASs was observed after rejuvenation. On day 30, ATP, and 2,3-DPG levels of AS-1 were consistently lower than AS-3 and AS-5.	120 days	[40]
SAGM, AS-1, AS-3, PAGGSM	CPD and CP2D	pH, sodium, potassium, chloride, pressure of CO_2,_ pressure of O_2_, glucose, glycolytic rate, intracellular citrate concentration, ATP, and 2,3-DPG levels.	Intracellular citrate concentrations were increased in RBCs stored in AS-3 and SAGM. A steady increase in the soluble O_2_ concentration was shown in SAGM and AS-1, and a decrease in sodium and an increase in potassium were observed in all solutions.	46 days	[41]
SAGM, AS-1	CPD	Hb levels, HCT, and hemolysis.	Hemolysis progressively increased from day 0 to day 42 in both solutions. A slightly lower level of hemolysis was observed in AS-1 compared to SAGM samples.	42 days	[42]
MAP, 0.9% NaCl	—	Free Hb, morphology, ATP, content of adenine nucleotides, and elongation index.	RBCs maintained a normal biconcave-disk shape after 4 h of preservation in both solutions, and some became acanthocytes after 4 h in 0.9% NaCl. ATP levels were significantly higher in MAP than 0.9% NaCl-stored RBCs. Hemolysis and free Hb increased over the storage time and were significantly higher in 0.9% NaCl.	4 h	[43]

**Table 3:** Effect of solution parameters on RBC preservation. These studies analyzed the effect of several storage parameters (first column) on RBC storage (sixth column).

Conditions Analyzed	Measured Parameters	Storage Solution	Anticoagulant	Storage Time	Main Results	Reference
Melatonin (MT) concentration	Morphology, RBC aggregation index, hemolysis, metHb, glucose, lactic acid, pH, malondialdehyde (MDA), and ATP level.	MAP	—	42 days	Deformation, relative hemolysis rate, aggregation index, MDA, and metHb were significantly affected throughout storage. The concentration of glucose, lactic acid, and ATP were affected by the storage time, but not by the MT concentration. The number of deformed RBCs, relative hemolysis rate, MDA, and metHb in the MT group were lower than that in the control group at the end of storage.	[54]
Temperature (−4 °C and 4 °C) and addition of either trehalose or polyethylene glycol (PEG) 400 (27.5, 55, 110, or 165 mM)	Hemolysis, deformability, ATP level, and RBC indices.	PAG3M	—	126 days	Adding PEG400 (110 mM) and keeping the units at −4 °C reduced hemolysis and improved deformability. The addition of trehalose increased hemolysis at −4 °C, even with adjustments to maintain the osmotic pressure. PEG400 performed worse with adjusted osmolarity.	[50]
Effect of glucose concentration (normal concentration: 111 mM for AS-1, 55 mM for AS-3, and 45 mM for AS-5; normoglycemic solutions with a concentration of 5.5 mM)	Glucose, Hb, ATP level, and RBC osmotic fragility.	AS-1, AS-3, AS-5, AS-1N, AS-3N, AS-5N	—	35 days	RBCs stored in normoglycemic solutions maintained their ability to release ATP, while those in standard solutions did not. RBCs stored under normoglycemic conditions (especially AS-1N and AS-5N) were initially less fragile than those in standard conditions.	[55]
Whole blood in CPDA-1, non-leukoreduced RBCs in CPDA-1, leukoreduced RBCs in CPD/SAGM	Hemolysis and EVs.	SAGM	CPDA-1, CPD	N/A	In CPDA samples, blood units with lower storage hemolysis (0.17% of hemolysis) had larger EVs, potentially able to enclose more Hb. Leukoreduced CPD/SAGM units showed a weaker correlation between hemolysis and EVs, suggesting the influence of mannitol and residual white blood cells/platelets on Hb distribution.	[56]
Addition of ascorbic acid (AA) in CPDA (2.06, 4.13, and 6.19 mg/mL)	EV production and coagulation time.	—	CPDA-1	35 days	Leukofiltration significantly reduced EV production. AA fortification showed a dose-dependent decrease in EV production. The highest AA concentration had the most significant EV reduction.	[51]
Effect of plasticizer (di(2-ethylhexyl) terephthalate (DEHT) and di(2-ethylhexyl) phthalate (DEHP))	Membrane stability, hemolysis, EVs, extracellular potassium, glucose, pH, lactate levels, MCV, and PS level.	SAGM, PAGGSM	—	28 days	DEHT with PAGGSM and DEHP with SAGM were equally affected up to 14 days post-irradiation for all parameters. For DEHT units, hemolysis and EV counts were increased at day 28, whereas extracellular potassium ions, glucose, lactate, pH, MCV, and EV PS remained unaffected. No individual unit exceeded 0.8% hemolysis. Membrane stability was least impacted in DEHP/PAGGSM.	[53]
γ-irradiation treatment	RBC aggregability, deformability, and EC interaction.	—	CPD	28 days	Cold storage elevated the number of adherent RBCs and the strength of their interaction with ECs and decreased RBC deformability. The RBC–EC interaction correlated with the translocation of PS to the RBC surface. γ-irradiation increased the number of rigid cells but did not affect adherence and aggregability.	[57]
Effect of plasticizer (DEHT and DEHP)	Hemolysis, potassium, glucose, lactate, ATP and 2–3,DPG levels, venous blood gas panel (pH, pO_2_, pCO_2_, and HCO_3_), and RBC morphology.	AS-1, PAGGSM	—	42 days	Hemolysis was higher in DEHT/AS-1 units. RBC morphology changed to a greater extent in DEHT with both AS-1 and PAGGSM, in comparison to DEHP units. No significant differences between DEHT- and DEHP-stored RBCs in AS-1 were noted for 2,3-DPG, pH, glucose consumption, lactate production, or potassium. ATP retention was >70% for all studied conditions.	[58]
Irradiation effect	Metabolomic profiles.	—	CPDA-1	35 days	Metabolomic profiles differed between fresh and old RBCs. Irradiation shifted profiles toward those of older cells, suggesting metabolic aging. Alterations in metabolites were related to cell membranes.	[59]
Effect of N-acetylcysteine (NAC)	Cell survival/hemolysis, glutathione, hydrogen peroxide metabolism, and peroxoredoxin-2 redox state.	SAGM	—	42 days	Higher NAC doses (20–25 mM) reduced hemolysis, but lower NAC concentrations prevented early glutathione loss. NAC partially preserved hydrogen peroxide metabolism but did not prevent peroxiredoxin alterations.	[60]
Effect of cold rejuvenation	Hemolysis, ATP, deformability, morphology, hematologic indices, blood gases, and potassium.	SAGM	CPD	49 days	Hemolysis, hematologic indices, pH, glucose, and potassium levels showed no difference between the tested conditions. ATP levels increased, deformability initially decreased, and morphology improved with the use of rejuvenation.	[61]
Effect of buffy coat	Hemolysis, potassium, and LDH.	SAGM	CPD	42 days	Hemolysis, potassium, and LDH were lower in SAGM units. No significant effect of buffy coat removal was observed.	[62]
Donor characteristics (gender) and RBC age	Hemolysis, morphology, deformability, metabolites, intracellular ROS, and calcium.	SAGM	CPD	28 days	Younger RBCs had better morphology, deformability, and hemolysis than older RBCs. Units derived from females had a slower increase in hemolysis compared to male donor units. Metabolomic differences were seen along with the heterogeneity of the RBC units for all conditions.	[63]
Effect of AA in storage solution	Mechanical fragility (membrane injury), hemolysis, blood gases (pO_2_ and pCO_2_), and metHb.	AS-5	—	42 days	AA reduced hemolysis and membrane fragility during storage. The addition of AA did not significantly alter RBCs’ biochemical parameters.	[64]
Mirasol treatment	Hemolysis, morphology, PS exposure, rigidity, redox changes, energy metabolism, and EVs.	SAGM	—	42 days	Mirasol pathogen-reduction treatment accelerated storage lesions and promoted eryptosis. Limiting the storage of Mirasol-treated RBCs to 21 days could help mitigate negative effects while retaining pathogen-reduction benefits.	[65]
Leukoreduction	LDH, lactate, glucose-6-phosphate dehydrogenase, Hb, hemolysis, and RBC indices.	SAGM	CPD	42 days	Leukoreduction helped reduce LDH, lactate, and hemolysis during prolonged storage compared to unfiltered units.	[66]
Rejuvenation temperature—standard (37 °C) or cold (4 °C)	Glycolytic metabolites, purines, glutathione, and fatty acids.	AS-1, saline	CPD	15 days	Compared to washing alone, standard and cold rejuvenation were more effective at improving energy metabolism and the glutathione status and preventing purine oxidation. Standard rejuvenation maximized the benefits related to energy metabolism. Cold rejuvenation presents potential operational advantages, priming energy and redox metabolism of even medium-aged, stored RBCs.	[67]
Blood unit segments sampling	Deformability, MCV, mean corpuscular hemoglobin (MCH), MCHC, and hemolysis.	—	—	56 days	The deformability of RBCs stored in blood bags was retained over 4 weeks, but a loss of deformability after that was observed. Strong correlations were found between the blood bag and segment for MCV, MCHC, and MCH but not for hemolysis.	[68]
Early γ-irradiation	Hb, ATP, EVs, PS exposure, and calcium levels.	SAGM	—	42 days	Early γ-irradiation accelerated hemolysis, ATP depletion, and EV release. Irradiated units showed increased susceptibility to stress-induced PS externalization after shorter storage periods (4–21 days).	[69]
Effect of irradiation and/or leukocyte filtration	2,3-DPG, pH, free Hb, potassium, sodium, MCV, MCH, and cell morphology.	MAP	—	35 days	Pre-storage treatments exacerbated the loss of 2,3-DPG, intracellular potassium leakage, and cell morphology changes over time compared to untreated units. γ-irradiation and leukoreduction may worsen RBC quality during subsequent refrigerated storage.	[70]
Delay (24 h) in unit preparation via leukoreduction	ATP, 2,3-DPG, lactate, pH, potassium, hemolysis, LDH, PS exposure, glutathione, catalase, superoxide dismutase, Hb oxidation, and MDA.	SAGM	CPD	42 days	Overnight storage of units at room temperature impacted ATP, 2,3-DPG, and hemolysis but did not significantly alter measures of oxidative stress or damage. Rapid cooling led to higher potassium levels early in storage. Metabolic changes were more important than oxidative damage.	[71]
Effect of liposome treatment	Hemolysis, potassium, RBC indices, deformability, aggregation, EVs, ATP, and 2,3-DPG.	HEPES-NaCl with/without 1,2-dioleopyl-sn-glycero-3-phosphocholine (DPOC)	—	42 days	DPOC liposome treatment modestly improved some hemorheological properties, like aggregation and rigidity, during storage but did not significantly alter metabolic parameters. Effects were more pronounced at early storage up to 3 weeks.	[72]
pH effects (8–9 for PAGGGM; 5–6 for SAGM)	PTR and metabolic recovery.	PAGGGM or SAGM	CPD	35 days	The longer storage time of RBCs was associated with a decreased PTR. PAGGGM storage showed a better metabolic profile but did not lead to a higher PTR.	[25]
Anaerobic conditions	% SO_2_, pCO_2_, metHb, ATP, and 2,3-BPG.	AS-3	CP2D	42 days	Storage lesions were % SO_2_ dose-dependent. The control of % SO_2_ may lead to a reduction in the adverse events associated with transfusion.	[73]
Cell age; anaerobic conditions	Rate of nitric oxide (NO) scavenging, morphology, MCHC, MCV, and membrane permeability.	AS-1	CPD	Fresh and old cells (8.5 vs. 37.5 days)	Old, stored RBCs scavenged NO 1.7–1.8 times faster than fresher RBCs. Biconcave geometry favored faster NO scavenging compared to spherical cells. A smaller MCHC or MCV led to increased NO scavenging.	[74]
Anaerobic conditions	Hb, oxidative status, and band 3 protein fragment.	SAGM	CPD	21 days	Two different degradation products of the cytoplasmic domain of band 3 were detected in RBC membranes during storage.	[75]
Multiple room temperature (RT) exposures; temperature 1–6 C and RT	Hemolysis, morphology, pH, and metabolism.	AS-3, SAGM	—	42 days	RT exposures increased hemolysis, especially in SAGM units. After 3 RT exposures, AS-3 units achieved 13% hemolysis, whereas SAGM units reached 27% hemolysis.	[76]
Anticoagulant effects	RBC count, pH, HCT, and Hb.	—	CPD, CP2D, and CPDA-1	35 days	The RBC count, pH, HCT, and Hb level did not significantly decline over time with any anticoagulant.	[77]
Anaerobic/aerobic storage	ATP, DPG, glucose, glyceraldehyde 3-phosphate, lactate, oxidative stress, PPP, and glutathione.	SAGM with CPD	CPD	42 days	Anaerobic storage promoted glycolytic metabolism, prolonging energy and purine reserves. Anaerobiosis impaired the RBCs’ ability to manage oxidative stress by preventing metabolic shifting to PPP, disrupting glutathione homeostasis; it caused less sustained oxidative stress than aerobic storage, but oxidative stress markers increased over time in anaerobically stored cells.	[78]
Anticoagulant and pH (7.4 or 8.2) effects	HCT, 2,3-DPG, ATP, lactate, hemolysis, intracellular pH, potassium, sodium, glucose, and extracellular pH.	PAGGGM, SAGM	CPD or trisodium citrate (TNC)	35 days	The pH of the anticoagulant used during whole blood collection affected the storage of RBCs. The pH of PAGGGM can be decreased to physiologic levels when an anticoagulant with a physiologic pH is used during whole blood collection, while maintaining ATP and 2,3-DPG levels.	[79]
Leukoreduction method and AS effect on neutrophils	Proinflammatory activity, hemolysis, and neutrophil priming activity.	AS-1, AS-3, AS-5	—	42 days	Filtration, buffy coat removal, and a combination of these two were tested. Filtration and combination leukoreduction decreased the accumulation of proinflammatory activity compared to the buffy coat method. The combination of methods was not more advantageous over filtration (increased costs and hemolysis). AS-3 decreased the early accumulation of neutrophil priming activity versus AS-1 or AS-5 during storage.	[80]
Anaerobic and aerobic storage and pH (6.5, 7.4, 8.3) effects	Cell count, free Hb, glucose, electrolytes, lactate, pH, ATP, 2,3-DPG, morphology, hemolysis, and PS.	AS-3, AS (6.5, 7.4, 8.3)	—	16 weeks	ATP and 2,3-DPG were better maintained in anaerobic storage than in aerobic storage. Acidic or neutral pH conditions preserved the ATP concentration better, but neutral or basic pH favored the maintenance of 2,3-DPG levels. AS pH had less of an effect on the exposure of PS, vesicle protein release, and hemolysis. The rejuvenation of RBCs during cold, anaerobic storage resulted in increases in ATP and 2,3-DPG levels and a reversal of PS exposure.	[81]
Effect of Mirasol treatment	Hematocrit, pH, hemolysis, Hb, p50, and potassium.	AS-3	—	21 days	Increased hemolysis, small differences in p50, and higher potassium levels were observed in Mirasol-treated samples. MetHb in Mirasol-treated samples was high after treatment but returned to normal levels within 24 h.	[82]

## Data Availability

Data are contained within the article.
